# Supplementary material for: Exploring new roles for actin upon LTP induction in dendritic spines
Source: Sci Rep. 2021 Mar 29;11:7072. doi: 10.1038/s41598-021-86367-z (PMC8007616; doi:10.1038/s41598-021-86367-z)
Supplement: Supplementary file 1 — Supplementary Information. [file 41598_2021_86367_MOESM1_ESM.pdf]

Exploring new roles for actin upon LTP induction in dendritic spines

Supplementary Information

Mayte Bonilla-Quintana[1,*] and Florentin Wörgötter[1]

[1]University of Göttingen, Germany.

[*]Corresponding author: mayte.bonilla-quintana@phys.uni-goettingen.de

Actin Force

Figure S1a shows the force generated by actin polymerization $\mathbf{F}_{actin}$ corresponding to Eq. (2) (see Methods) in a spine at different times. This force generates a protrusion that moves at a decreasing velocity (Fig. S1b). Note that ϕ in Eq. (2) is inversely proportional to the number of polymerization foci so that spines with different number of polymerization foci have a similar amount of $\mathbf{F}_{actin}$ at the start of the simulation. Figure S2 illustrates how the simulation behaves if we use a constant value of ϕ. Note that the spine with 22 foci and $\phi = \phi(n_{f=1})$ increases its volume to v^* rapidly but the maximum value of the force generated by the membrane tension is lower than that of the spine with one focus and $\phi = \phi(n_{f=1})$.

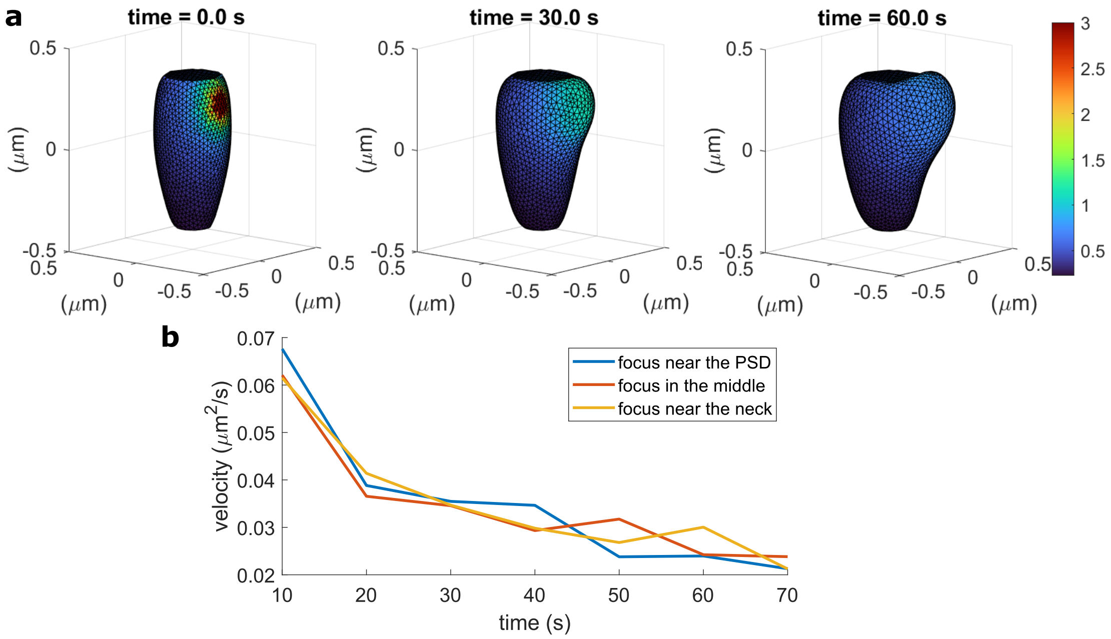

Figure S1: **Force generated by actin polymerization**. **a**, snapshot of spines at different times. Color denotes the amount of force generated by actin polymerization located in a focus near to the PSD. Note that the actin force decreases as the spine grows. **b**, evolution of the velocity over time. Here, the velocity is calculated using the tracking point $\mathbf{x}$ closest of the membrane at the start of the simulation. The velocity is equal to the displacement of this tracking point every ten seconds, i.e., velocity$(time = t) = ||\mathbf{x}(t) - \mathbf{x}(t-10)||$, $t = 10, 20, \ldots, 70$. The data correspond to the simulations in Figure 3.

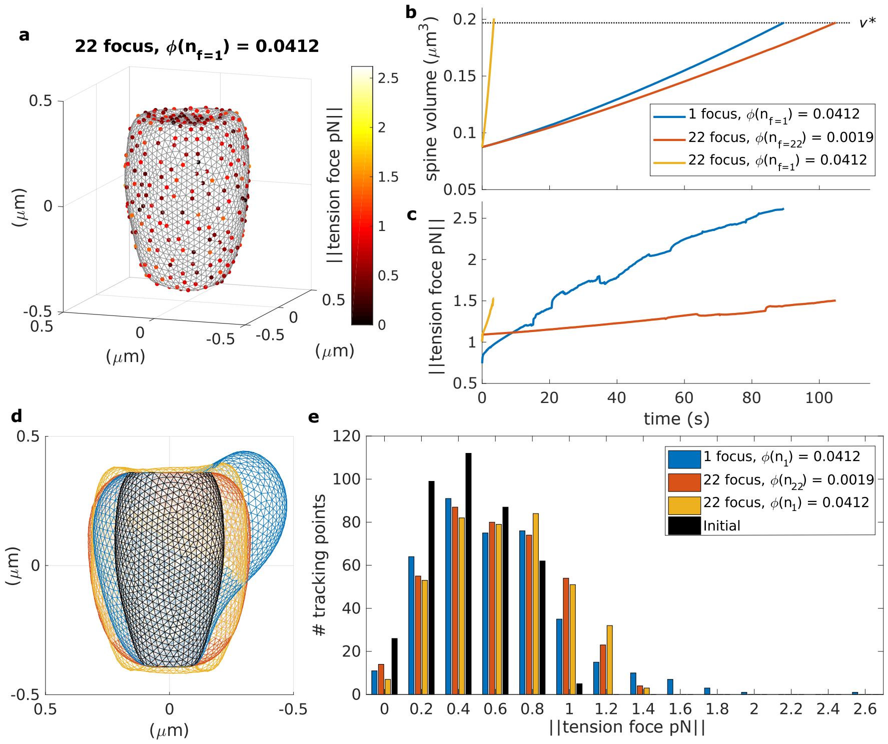

Figure S2: **Spine enlargement upon LTP for different locations of the F-actin polymerization foci and ϕ.** **a**, shape of a spine with 20 equally distributed polymerization foci and $\phi(n_1) = 0.0412$ (gray) at time when it reaches a volume of v^*. Dots are the tracking points color-coded for the membrane tension force. **b**, spine volume evolution over time, color-coded for different parameters. Dotted black line denotes v^*. **c**, the evolution of the membrane tension for the tracking point with maximum membrane tension when the spine reaches a volume of v^*. **d**, spine shapes for different parameters when they reached a volume of v^*, color-coded as in (b). This plot shows the $y - z$ axis, slid at $x = 0$. Black shape corresponds to the resting shape. **e**, histogram of the distribution of the force generated by membrane tension measured at the tracking points for the spines in (d).

Results Validation

Next, we present additional simulations to verify our results. Figure S3 shows that the sum of the force generated by membrane tension is significantly higher in spines with polymerization focus near to the PSD (according to a Mann-Whitney U-test p-val < 0.05). The results of Figure S4 are similar to those of Figure 3 despite using different number of evenly distributed tracking points.

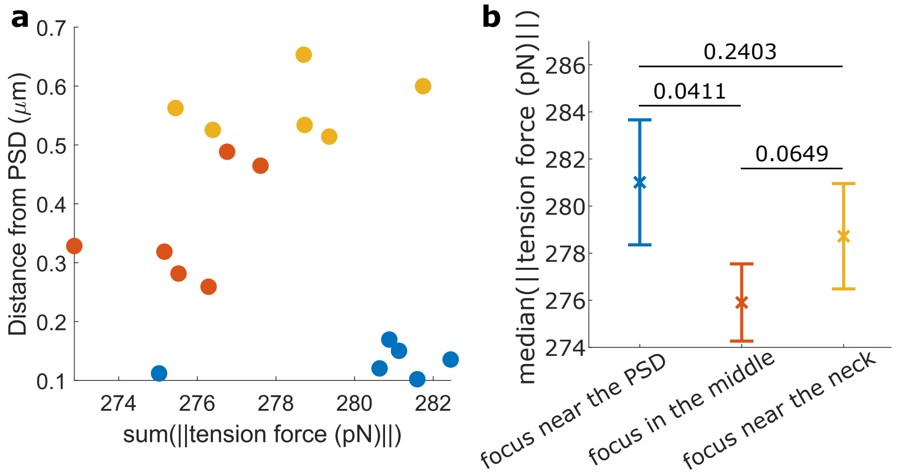

Figure S3: **Sum of the force generated by membrane tension**. **a**, the distance of the actin polymerization focus from the PSD against the sum of the force generated by membrane tension when the spine reaches a volume of v^* for different simulations. The foci were randomly allocated. **b**, median $\pm$ standard deviation of the sum in (a) for different regions of the spine. Numbers are the p-values of a Mann-Whitney U-test.

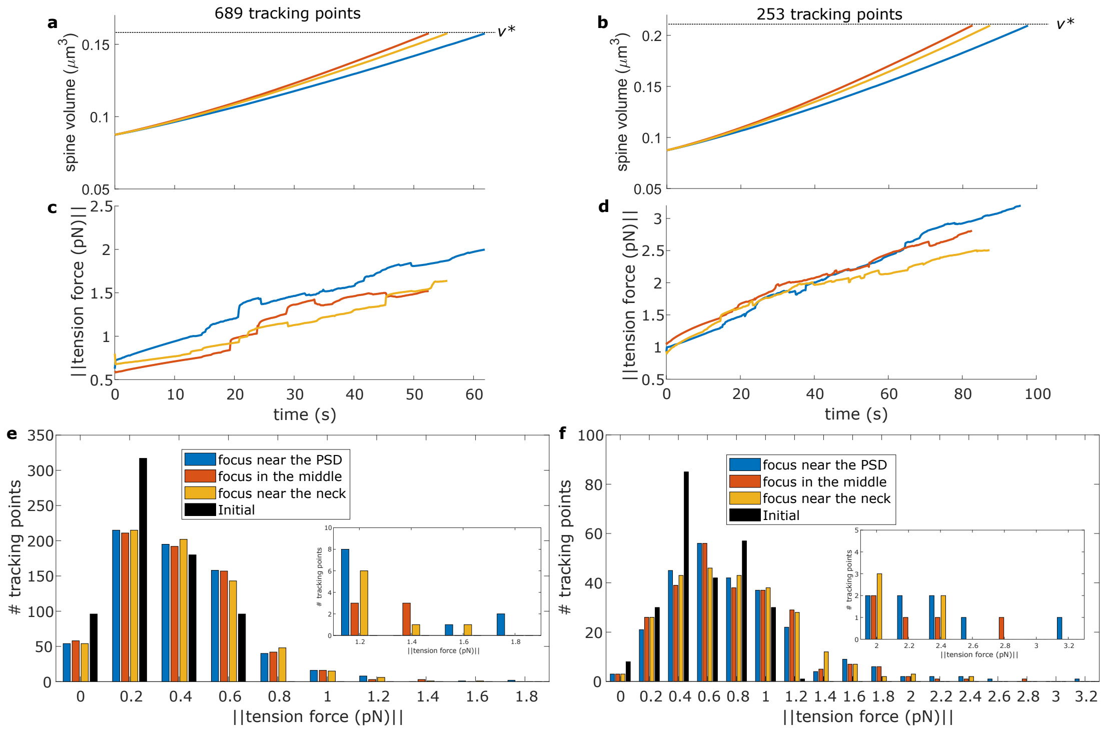

Figure S4: **Simulation with different set of tracking points**. Simulations corresponding to 689 (left) and 253 (right) tracking points. **a-b**, spine volume evolution for spines with polymerization focus at different locations, color-coded as in (c). v^* corresponds to the volume when the spine with one actin polymerization focus increases its tension 2.5 fold. **c-d**, evolution of the tension measured by the tracking point with the higher tension when the spine reaches a volume of v^*. **e-f**, histogram of tension measured by the tracking points when the spine reaches a volume of v^*.
